# Urinary tract infections attributed to diverse ExPEC strains in food animals: evidence and data gaps

**DOI:** 10.3389/fmicb.2015.00028

**Published:** 2015-02-04

**Authors:** Randall S. Singer

**Affiliations:** ^1^Department of Veterinary and Biomedical Sciences, University of Minnesota, Saint Paul, MN, USA; ^2^Instituto de Medicina Preventiva Veterinaria, Facultad de Ciencias Veterinarias, Universidad Austral de Chile, Valdivia, Chile

**Keywords:** *Escherichia coli*, antibiotics, antibiotic resistance, urinary tract infections, UPEC, APEC

## Abstract

Between 70 and 95% of urinary tract infections (UTI) are caused by strains of *Escherichia coli.* These strains, often termed Extraintestinal Pathogenic *E. coli* (ExPEC), possess specific virulence traits allowing them to colonize more inhospitable environments, such as the urogenital tract. Some ExPEC isolates from humans have similar virulence factor profiles to ExPEC isolates from animals, and because of the potential for these strains to cause UTI in people, these infections have been referred to as foodborne UTI, or FUTI. Finding similarities in ExPEC in animals and humans is not necessarily proof of transmission, particularly a unidirectional pathway from animals to humans; similarities in virulence factor profiles should be expected given the specific bacterial requirements for colonizing physiological compartments with similar characteristics in all animals. Many of the most important strains of human ExPEC globally, such as ST131, are highly virulent and clonal implying routes of transmission other than food. Documenting routes of transmission is particularly difficult due to the wide range of potential ExPEC sources, including the human intestinal tract, and non-human reservoirs such as food animals and retail meat products, sewage and other environmental sources, and companion animals. The significant environmental reservoir of ExPEC, including strains such as ST131, could potentially explain much more completely the global dissemination of virulent ExPEC clones and the rapid dissemination of new strains within the community. Taken in its totality, the link between ExPEC in animals and UTI in humans might exist, but studies conducted to date do not enable an estimation of the relative importance of this route of transmission. To reduce the burden of illness associated with ExPEC, the scientific community needs to push forward with ecologically-based, scientifically-sound study designs that can address the plethora of ways in which *E. coli* can spread.

## INTRODUCTION

Extraintestinal pathogenic *Escherichia coli* (ExPEC) is an incredibly diverse *E. coli* pathotype, and this genetic diversity is reflective of its occurrence in and colonization of diverse and highly specialized ecological niches. These strains, which can reside in the gastrointestinal (GI) tract, differ from normal commensal strains in that they possess virulence traits that allow them to colonize more inhospitable environments, such as the urogenital tract (Smith et al., 2007). Aside from these bacteria-specific traits, host-specific factors are also required for disease. For this reason, ExPEC is considered a necessary but not sufficient cause for extraintestinal *E. coli* infection, meaning that additional factors are required for illness to occur (Rothman et al., 2008). Consequently, ExPEC is considered an opportunistic pathogen for causing ExPEC-related illness.

In general, ExPEC can colonize the human intestine, and when given the opportunity in individuals who might be vulnerable in some way (e.g., compromised immune system) or the presence of specific risk factors such as increased sexual activity and use of spermicides (Foxman, 2014), the bacterium can be transferred to the urogenital tract where it can cause a urinary tract infection (UTI). UTIs represent a serious burden of illness globally (Foxman, 2002, 2014), and 70–95% of these infections are caused by strains of *E. coli* (George and Manges, 2010; Hooton, 2012), often termed uropathogenic *E. coli* (UPEC), which is a subset of ExPEC. Although antibiotic treatment is only necessary in some cases of UTI, antibiotic resistant infections can increase the costs associated with treatment (Alam et al., 2009). Resistance to antibiotics that are important for treating UPEC infections has been steadily increasing (Nordstrom et al., 2013).

The time between GI colonization and subsequent extraintestinal infection can be lengthy, with some studies using a 6-month risk window for development of the UTI (Manges et al., 2007). This extended timeframe between exposure to an ExPEC strain and subsequent illness makes it very challenging to establish a “source” for the strain given the constant ebb and flow of *E. coli* strains within human and animal GI tracts. In a classic study of *E. coli* variability, *E. coli* populations in the GI tract of the human host changed frequently over time through clonal replacement, but the ecological and genetic reasons for these changes were never clarified (Caugant et al., 1981). Similar observations of a high rate of *E. coli* turnover in the GI tract have also been made in animal populations (Hinton, 1986; Hinton et al., 1986).

It is well-accepted that bacteria such as *E. coli* can move passively to humans through a variety of routes, including the food chain. Because of the similarity between some human and animal ExPEC and the potential for these strains to cause UTI in people, these infections have recently been referred to as foodborne UTI, or FUTI (Nordstrom et al., 2013). However, finding similarities in ExPEC in animals and humans is not necessarily proof of transmission, in general, or in a unidirectional pathway from animals to humans, specifically. Similarities in strains isolated from different host species should be expected, particularly with respect to specific virulence factors (VF). These VF are important to ExPEC survival in these extraintestinal environments. Furthermore, while it is commonly assumed that the use of antimicrobials in animal agriculture is the primary cause of antibiotic resistance (AR) observed in many human UPEC strains, the presence of AR traits within these bacteria is not proof of a cause and effect relationship with antimicrobial use in animal agriculture. Overall, phenotypic trait typing may not reflect underlying genetic dissimilarity, and even in the event of documenting identical genotypes among hosts, a common source cannot be determined without appropriate study design.

A continued reliance on phenotypic traits, such as VF and AR, and the use of study designs inappropriate for documenting frequency and direction of transmission, has resulted in the propagation of the ill-defined inference that animal ExPEC cause a substantial fraction of human ExPEC infections, including UTI. This review will explore the complexities of interpreting ExPEC-related data with respect to distribution, illness and transmission. The attribution of ExPEC-associated UTIs to specific sources, including the potential for a causal link between animal ExPEC and human UTI, will be discussed. Finally, this review will highlight study design issues that should be considered to provide better insight into the serious global burden of ExPEC-related illness.

## UPEC AND UTI IN THE COMMUNITY

Urinary tract infections are generally considered sporadic illnesses within the community. Some researchers believe that there have been outbreaks of UTIs within communities (George and Manges, 2010), including south London (Phillips et al., 1988), Copenhagen (Olesen et al., 1994), Calgary (Pitout et al., 2005), and California (Manges et al., 2001). These case series, which often involve a diverse set of strains within a clonal group, lack a clear definition of what constitutes an outbreak. For example, in a purported outbreak of UTIs caused by ExPEC in clonal group A (CgA), which as a group can lead to bacteremia (Skjot-Rasmussen et al., 2013), the isolates actually belonged to four different serotypes (Manges et al., 2006). Infection in the community with commonly circulating ExPEC strains is normally not a sufficient outbreak definition, particularly with an opportunistic pathogen, if the overall burden of illness (incidence of UTI within the community) does not differ from the expected rate. As stated in a publication regarding outbreak defintions, genotype data are used to support the epidemiolgoical data in defining an outbreak (Riley and Manges, 2005), but simply having a common set of genotypes in a specific time and place is not sufficient for defining an outbreak with an opportunistic pathogen.

Reports of purported UTI outbreaks have been interpreted as being consistent with the introduction of these new strains into the community through a single point source, such as food. However, these reports are also consistent with regular, broad clonal shifts in the endemic ExPEC strains within the community. When data on the predominant UPEC strains within the community are assessed over time, newly identified strains can become endemic and then persist for extended temporal periods (Manges et al., 2008). A study in a single community (Smith et al., 2008) noted shifts in the predominant UTI-associated strains over time, a finding that might be more consistent with horizontal transmission or widespread exposure to a common source such as water rather than a limited food item. It would be plausible to have a strain introduced via a limited food vehicle such as meat and then disseminated more broadly via human-to-human or environment-to-human transmission. Regardless, this constant turnover in *E. coli* should not be considered synonymous with continual outbreaks linked to novel strains.

Because of the potential to have some of these strains introduced into a community via food, some investigators have stated that a potential common source of UPEC is analogous to an *E. coli* O157:H7 outbreak (Manges et al., 2006). *E. coli* O157 and other EHEC strains cause severe GI infection soon after ingestion; extended colonization of the healthy human host with these strains is rare as is person-to-person transmission. This situation is very different from UTIs caused by ExPEC that colonize the healthy human, where person to person transmission may be the primary mode of movement of these strains. These situations are not analogous, and comparing them can be misleading. Applying outbreak definitions from organisms like *E. coli* O157 to ExPEC-related UTIs is entirely inappropriate.

Of considerable and justified concern is the global dissemination of highly virulent ExPEC clones over the past decade (Petty et al., 2014; Riley, 2014), including the clonal complex known as sequence type 131 (ST131) which now causes the majority of extraintestinal infections in humans, including UTI (Nicolas-Chanoine et al., 2008; Novais et al., 2012; Price et al., 2013). This clonal complex is thought to be highly virulent and is commonly multidrug resistant, including resistance to the critically important antibiotic classes of fluoroquinolones and third and fourth generation cephalosporins. The transmission pathways for these clones to disseminate globally remain unclear. The ST131 pandemic in humans appears to have little relationship to animal agriculture, at least in its origins, and likely emerged through evolutionary pressures in human hospitals and the community (Platell et al., 2011b; Johnson et al., 2012a). This strain is also found in companion animals, including pets that share residence with humans (Ewers et al., 2010; Platell et al., 2010, 2011a; Osugui et al., 2014) and has been documented to spread among dogs and cats within the household (Johnson et al., 2009d). While the possibility for horizontal transfer of virulence genes among *E. coli* exists (Johnson and Nolan, 2009), the global distribution of these highly virulent clones in multiple hosts and clear evidence of direct horizontal transmission among hosts would appear to make food an unlikely vehicle by which to explain this pattern of disease.

## HUMAN AND ANIMAL EXTRAINTESTINAL *E. coli*

The establishment of extraintestinal disease by ExPEC is complex, usually involving strains carrying necessary VF first colonizing the GI tracts of individuals at-risk for disease (Moreno et al., 2008). There are no concrete criteria, however, for defining an *E. coli* strain as ExPEC. Some groups have made the assumption that possessing at least two of the following genetic determinants warrants the ExPEC label: *papA* and/or *papC* (P fimbriae structural subunit and assembly), *sfa/focDE* (S and F1C fimbriae), *afa/draBC* (Dr binding adhesins), *iutA* (aerobactin system), and *kpsM* II (type II capsule; Johnson et al., 2007a). This minimal predictive set of ExPEC virulence genes is based upon genotyping of many virulence-associated genes, but this subjective definition has not been thoroughly validated, as strains meeting this definition do not always cause disease. Using a list of known VF as a criterion misses the remaining genes in the pathogen’s genome that may also be required for disease, but these might not be in all strains with the “correct” VF distribution.

Given the fact that ExPEC require specific VF to cause disease in animals, it should be expected that there will be similarities in ExPEC across animal species. This is particularly true if the total array of possible VF is reduced to the minimal predictive set described previously. The niches that the ExPEC strains colonize extraintestinally require specific bacterial adaptations, and because some of these requirements are similar across animals, similarities in the subsets of VF necessary for causing extraintestinal disease in different animal hosts are expected. Among *E. coli* that cause extraintestinal disease in poultry, also known as avian pathogenic *E. coli* (APEC), similarities in VF profiles have been observed with the human ExPEC strains (Rodriguez-Siek et al., 2005; Johnson et al., 2008a; Belanger et al., 2011; Danzeisen et al., 2013). Similarity in VF profiles of ExPEC from companion animals and humans has also been observed (Johnson et al., 2009a).

Extraintestinal Pathogenic *E. coli* from both animals and humans have been shown to have the ability to cross host species barriers. In mouse models, APEC and human ExPEC strains were shown to have UTI-causing ability (Jakobsen et al., 2010a, 2012). Neonatal meningitis caused by *E. coli* (NMEC) is a serious and often fatal disease. In rat models, APEC strains were capable of causing meningitis (Tivendale et al., 2010), although considerable work still needs to be performed to fully understand virulence requirements in these isolates (Logue et al., 2012). Human ExPEC strains have also been shown to be virulent for chicks (Moulin-Schouleur et al., 2007). Continued research in the area of VF is needed to determine specific VF required for disease in different hosts, factors that select for increasing virulence, and the frequency with which ExPEC isolates cross the host-species barrier in the real world. This topic will be discussed further in the Source Attribution section below.

## ANIMAL ANTIBIOTIC USE, ANTIBIOTIC RESISTANCE, AND THE RELATIONSHIP TO HUMAN UTI

Perhaps the most confounding aspect of the discussion of human UTI related to foodborne transmission and animal agriculture is the issue of antibiotic use and resistance. The concern over antibiotic resistant strains of UPEC is warranted given that resistance to the antibiotics of choice for treating human UTIs has been observed and some compounds are no longer prescribed at all because of high resistance rates (Nordstrom et al., 2013). A number of studies have documented similar AR traits of contemporaneously sampled human and animal ExPEC (Johnson et al., 2007a; Hancock et al., 2009; Jakobsen et al., 2010b; Bergeron et al., 2012) and have attributed the resistance to antibiotic use in agriculture. None of these studies has ascertained antibiotic exposure information and none has used analytical approaches that would aid in the determination of the origins of resistance. With findings of identical resistance profiles in *E. coli* sourced from antibiotic-free meat sources (Johnson et al., 2007a) and the absence of data on antibiotic use from other meat sources, these study designs do not enable conclusions regarding the origins of the resistance or the selection pressures that may have contributed to the observed resistance. The presence of similar AR phenotypic patterns is insufficient for documenting likely transmission, as discussed below in the Source Attribution section.

The focus of most discussion concerning antibiotic resistant ExPEC in animals has been on antibiotics used for growth promotion/feed efficiency (Nordstrom et al., 2013; Riley, 2014). The antibiotics approved for this use, at least in poultry, have little activity against *E. coli* or are unrelated to the resistances that are relevant for treatment of human UTIs. Trimethoprim and sulfamethoxazole are antibiotics important for treating human ExPEC infections, but neither is used for growth promotion in the U.S. Fluoroquinolones such as ciprofloxacin are also important for treating human UTIs; these compounds have never been approved for growth promotion and were withdrawn from use in U.S. poultry in 2005. In fact, the National Antimicrobial Resistance Monitoring System (NARMS) in the U.S. has only identified two *E. coli* isolates from chicken meat with ciprofloxacin resistance since 2002 (U.S. Food and Drug Administration, Center for Veterinary Medicine, 2012b). The prevalence of ciprofloxacin resistance in UPEC has been steadily increasing, with the rate apparently accelerating even after the withdrawal of fluoroquinolones from U.S. poultry (Sanchez et al., 2012). Enrofloxacin is used in companion animals, and enrofloxacin resistance in *E. coli* UTI in dogs has been documented (Cooke et al., 2002). Although the isolates from this study had VF patterns that differed from the most common human ExPEC isolates (Johnson et al., 2009a), transmission between humans and companion animals is well-documented, as described previously.

Within the U.S., the U.S. FDA Center for Veterinary Medicine has initiated the process of phasing out of the growth promotion/feed efficiency label for critically important antibiotics over a 3-year period, to be completed by December 2016 (U.S. Food and Drug Administration, Center for Veterinary Medicine, 2012a, 2013). Consequently, while more research is needed to clarify the relationships between remaining on-farm antimicrobial usage and AR in human pathogens, a continued focus on the growth promoters will prevent us from accurately understanding and mitigating the pandemic crisis of multidrug resistant ExPEC.

Resistance to critically important antibiotics can be frequently found in ExPEC isolates, including UPEC and APEC. Similarities in the VF profiles of resistant and susceptible strains from animals have been interpreted as evidence that resistance develops in animals because of antibiotic use and is then transferred to people (Johnson et al., 2007a, 2009b). The conclusions reached in the study by Johnson et al. (2007a), in particular, are problematic and not necessarily supported by the data and yet these conclusions have been widely cited, including in high profile documents such as the recent President’s Council of Advisors on Science and Technology (PCAST) report from the Executive Office of the President of the United States (President’s Council of Advisors on Science and Technology [PCAST], 2014). For example, in that study both resistant and susceptible poultry isolates differed from the human isolates. The principal coordinates analysis that was based on virulence genotypes and phylogroups showed this distinction. Because the authors felt that the resistant human isolates were more similar overall to the poultry isolates than to the susceptible human isolates, they concluded that the resistant strains in humans must have been derived from poultry. Furthermore, the finding of similar resistant strains in meat-eaters and vegetarians was interpreted as consistent with human-to-human transmission or errors in reporting of poultry consumption rather than human strains being derived from a source other than chicken. If these illnesses were truly being sourced from poultry, one would expect to see the susceptible human and poultry isolates overlapping as well, a finding not supported by this study. Finally, the observation that resistant and susceptible poultry ExPEC isolates were similar with respect to virulence should indicate that resistance is unlinked from virulence, likely through the gain and loss of resistance-encoding plasmids. This is not synonymous with the conclusion that resistance develops in poultry and subsequently spreads to humans. While it is possible for the virulence traits to be co-located with AR traits within the bacterium, in avian-source strains it is more common to observe virulence-associated traits on a plasmid that has no resistance traits (Johnson et al., 2012b).

## SOURCE ATTRIBUTION

It has been stated that to “establish a link between human and animal ExPEC strains, isolates need to be compared” (Belanger et al., 2011). However, simply finding similarities between animal and human isolates is not the same as documenting the routes of transmission and the frequency by which strains from animals colonize humans and cause subsequent disease. While the transmission of ExPEC among differing hosts is biologically plausible given the overlap in VF, this is different than performing the more arduous task of quantifying the frequency and directionality of these events. One way to think about source attribution from a causal perspective, for example between chicken consumption and UTI, is to ask the question “if no more chicken were consumed, how many *E. coli* UTIs would be prevented and how much would the prevalence decline?” Note that this statement sounds similar to the commonly used epidemiological attributable fraction. The attributable fraction, however, is frequently misinterpreted; it is typically based on observational data and most often represents a statistical association and not a causal relationship.

Documenting routes of transmission is particularly difficult due to the wide range of potential ExPEC sources, including the human intestinal tract, and non-human reservoirs such as food animals and retail meat products, sewage and other environmental sources, and companion animals (Manges and Johnson, 2012). To identify the important sources and routes of transmission and to quantify the frequency of these transmission events, more rigorous epidemiological methods are needed, including improvements in study design, data analysis and data interpretation in addition to the incorporation of better molecular methods such as whole genome sequencing (WGS). A recent paper using *E. coli* ST131 as a model demonstrated a novel approach that might aid in identifying sources and routes of transmission of ExPEC strains (Lanza et al., 2014), with broad applications discussed in the accompanying perspective paper (Achtman and Zhou, 2014). Given that we can only attribute illnesses to sources included in the study, more attention needs to be given to the inclusion of diverse sources in overlapping spatial and temporal sampling frames. Study designs that enable the assessment of human-to-human and environment-to-human transmission must be utilized.

In general, most studies that have characterized ExPEC isolates from humans, both diseased and healthy, and from animals and animal meats have found that the animal isolates did not have the exact complement of virulence genes detected in the isolates from UTIs or the community samples (Ewers et al., 2007; Jakobsen et al., 2010c; Kluytmans et al., 2013). APEC strains in particular have some of the same VF as UPEC strains (Johnson et al., 2008a; Danzeisen et al., 2013; Mora et al., 2013; Maluta et al., 2014), but when a broad range of VF is assessed, considerable differences in the VF array of these ExPEC lineages become apparent (Johnson et al., 2008b; Belanger et al., 2011). This discrepancy has been acknowledged as consistent with the presence of other sources of ExPEC virulence genes (Ewers et al., 2007) and could reflect convergence on a similar set of virulence traits required by ExPEC in different animal hosts to colonize specialized niches. Some of these studies included methods such as pulsed-field gel electrophoresis (PFGE), multilocus sequence typing (MLST), and suppression subtractive hybridization (SSH) in addition to VF profiling and found that, while the isolates were similar, there was a clear distinction between the avian and human subpopulations (Moulin-Schouleur et al., 2006, 2007; Kariyawasam et al., 2007).

Although many studies have used VF and AR profiles to infer patterns of transmission, these approaches are inadequate for establishing transmission events of ExPEC. These methods lack the necessary resolution for establishing actual similarity of ExPEC strains among hosts. Similarities in VF and AR in ExPEC isolated from different animal species could indicate (1) unidirectional transmission from one species to another, (2) bidirectional transmission between the species, (3) transmission to each species from a common external source, or (4) completely independent pathogen transmission pathways, as has been described in specific subsets of *Salmonella enterica* (Mather et al., 2012, 2013). In particular, the analysis by Mather et al. (2013) that applied WGS to *Salmonella* Typhimurium DT104 isolates collected contemporaneously from animals and humans in Scotland showed distinct bacterial populations in humans and animals, thus questioning the dogma that this strain was associated with an epidemic spreading from animals to people. These types of molecular approaches and analyses are needed to provide better resolution to the complex ecology of ExPEC (de Been et al., 2014). A recent study using ST131 as a model highlighted one approach that might help clarify the complex distribution and long-distance dissemination of ExPEC strains such as ST131 (Lanza et al., 2014).

As WGS becomes more commonly employed in epidemiological investigations it will become easier to quantify the likely overlap of ExPEC populations among different hosts. An early study using WGS on ExPEC strains found that a prototypical APEC strain (APEC O1:K1:H7) had over 95% sequence identity with many human ExPEC whole genome sequences (Johnson et al., 2007b). A more recent study further demonstrated this high genomic similarity and showed the ability of APEC strains to cause meningitis in a neonatal rat model (Zhu Ge et al., 2014). While these results support the notion that ExPEC in animals and humans are highly similar, differences of 5% at the genomic level would not be suggestive of a recent transmission event.

In the 1990’s, as PFGE was gaining increasing usage as a method for typing bacterial isolates, criteria were established for interpreting relatedness of isolates collected during an outbreak and for helping determine if isolates were likely part of the same outbreak. These relatedness criteria put forth by Tenover et al. (1995) have been widely used for linking isolates to an outbreak strain and for suggesting plausible transmission events. Criteria such as these are needed for interpreting the comparative genomic analyses that incorporate WGS. For ExPEC isolates collected from multiple sources in a contemporaneous and spatially overlapping sampling frame, a better understanding is needed regarding the expected number of single nucleotide polymorphisms (SNPs) if the isolates were, in fact, transferred from one source to another. Evolutionary analytical tools well established in species ecology, such as phylogenetic diffusion modeling, have been successfully employed to draw inference about the relatedness of host-specific bacterial pathogen populations over time (Mather et al., 2013). Making decisions after the study about the degree of inter-strain similarity that might constitute source overlap and then making subjective interpretations of potential transmission based on these *post hoc* decisions will only continue the propagation of biased conclusions relative to ExPEC ecology and disease.

In addition to improving the laboratory and analytical approaches for studying ExPEC, improvements are needed in ExPEC study designs. Many studies involving ExPEC assume *a priori* that animals are the source of the ExPEC strains infecting humans and therefore have used a study design that oversamples specific animal commodities. These studies also tend to assume that antimicrobial resistance is the key trait of interest and therefore include oversampling of isolates that possess these resistance traits. By restricting the sampling scheme to very few sources, and by using biased oversampling techniques, it becomes impossible to estimate possible source attribution and the relative importance of one source versus another. These biases are further compounded by the continued use of typing methods that lack specificity, such as the exclusive reliance on VF and AR profiles.

As an example, a series of studies by one research group oversampled resistant isolates and restricted the range of possible sources to primarily chicken, beef and pork (Vincent et al., 2010; Bergeron et al., 2012). The studies presented no data about individual UTI cases, including the many exposure factors that increase one’s risk for developing a UTI. Although there were considerable differences between the isolates collected from UTIs and meats, the authors found that more isolates from chickens were in the same clonal groups with UTI isolates than beef cattle and pigs and thus concluded that this “confirms our hypothesis that chickens are a likely reservoir for ExPEC in humans” (Bergeron et al., 2012, p. 420). To properly evaluate the possible role of food as a source of human UPEC, finding broad similarities in the genetic profiles of isolates is insufficient to demonstrate transmission and source attribution.

The *a priori* assumption that meat is the source of human ExPEC is used to justify another study design: the comparison of ExPEC in meat-eaters and vegetarians. This type of comparison is typically biased because of the extreme confounding that exists; in other words, there is the implicit assumption that differences in the GI bacteria of these two groups can be attributed to the sole factor of meat consumption. When assessing the individuals enrolled in these studies, they often differ by many significant factors, including age and health status, and one early study found that hospitalization was significantly more important than diet in predicting the presence of resistant GI bacteria (van den Braak et al., 2001). In a study that was described above (Johnson et al., 2007a), the similar ExPEC strains in the healthy vegetarian comparison group and the hospitalized human group suggests a common non-meat source or significant human-to human transmission. The authors concluded, however, that individuals have trouble recalling poultry exposures and that the isolates were likely poultry-derived and either ingested inadvertently by the vegetarians or transmitted from meat-eaters to vegetarians. To evaluate the importance of animal sources to human ExPEC, better comparison groups are required with more detailed exposure histories recorded throughout the longitudinal investigation.

Perhaps the greatest challenge in understanding the sources of human UPEC is determining directionality of transmission of these strains. Most papers studying FUTI assume unidirectional movement of ExPEC isolates from animals to humans via the meat supply, even though the importance of human-to-human transmission is often the best explanation for certain infection patterns (Johnson et al., 2009c). Realistically, the movement of these strains likely includes complex environmental dissemination routes rather than unidirectional from one host species to another. The global dissemination of highly virulent ExPEC clones is a clear testament to these more complex transmission pathways (Petty et al., 2014; Riley, 2014). When WGS is applied to *E. coli* from many sources, including natural environments, our understanding of natural habitat, physiological function, and sources for potential transmission will undoubtedly change (Luo et al., 2011).

Given that most *E. coli* UTIs begin with colonization of the human intestinal tract by these ExPEC strains before infection of the urogenital tract can occur, there will be extensive human shedding of these ExPEC isolates into the environment via feces. Studies have shown that *E. coli* with uropathogenic VF can survive the sewage treatment plant process (Anastasi et al., 2010) and can be found in the waters surrounding these sites (Anastasi et al., 2012). ESBL-producing *E. coli*, including ST131, have been found in wastewater treatment plant effluent, demonstrating a clear environmental loading due to anthropogenic influence (Amos et al., 2014). As stated in this study, “Whilst it is not possible to determine the direction of spread from humans to animals, the significant environmental reservoir in rivers will impact both.” Some authors have acknowledged the potential role of water as a source (Belanger et al., 2011), and this could help explain the finding of antibiotic resistant *E. coli*, including ST131, in wildlife (Tausova et al., 2012; Hasan et al., 2014). This significant environmental reservoir could potentially explain much more completely the global dissemination of virulent ExPEC clones and the rapid dissemination of new strains within the community. To untangle the complexity of ExPEC ecology, we must do a better job at study design and recognize the broad range of potential sources and routes of transmission. Studies that incorporate proper study design and WGS, such as the recently published paper on ST131, are desperately needed (Lanza et al., 2014).

## CONCLUSION

Without doubt, there is potential for ExPEC to be transferred to humans as a result of the mishandling and/or undercooking of meats. Inadvertent ingestion of these strains leads to the potential for colonization of the intestinal tract, and if conditions are right, these strains can then gain entry to the urinary tract in compromised individuals. The likelihood and frequency of this occurrence, though, has not been demonstrated.

One approach that would enable the blending of the ecological and epidemiological aspects of ExPEC and provide a mechanism by which to investigate the likelihood and frequency of transmission events is through the integration of modeling efforts with empirical epidemiology and microbiology in an iterative process (Restif et al., 2012). This “model-guided fieldwork” framework, originally proposed in the context of wildlife ecology, dictates that models should be constructed early and inform all stages of the investigative effort from generation of hypotheses through the design of experimental trials and data analysis. Models resulting from this framework are data driven, specific to the system under study and able to provide highly useful insights. The end result of a study designed and performed under this framework is one that encompasses a range of plausible transmission routes, has appropriate sampling frames, and utilized laboratory methods in a manner that facilitates objective inferences about evolution, transmission and attribution.

As we develop a more ecological perspective to understand ExPEC transmission, there is a critical need to eliminate confirmation bias and subjective scientific inference. Confirmation bias is the reporting of information or the interpretation of equivocal results as supporting one’s *a priori* beliefs at the expense of alternate and equally plausible options (Borrell-Carrio and Epstein, 2004; Cox and Popken, 2008). In the case of FUTI studies, this would result in authors concluding that animals are the likely reservoir of human UTIs, without considering the totality of data and by excluding important alternative transmission pathways. Subsequent studies are designed around this “fact,” thus biasing any future inferences. As stated in one paper, “the present study population, which consisted of resistant *E. coli* only, may have been biased toward chicken-source isolates, which predictably would confound the ability of discriminant analysis to accurately predict the true original source of the isolate” (Kluytmans et al., 2013). Sound ecological investigations of ExPEC that account for many different possible sources and many different possible transmission routes are desperately needed.

The genetic basis of virulence in the ExPEC lineage that has commonalities in animals and humans, and the potential for animal ExPEC to cause disease in humans, cannot and should not be ignored. The reality of ExPEC is that there is likely some intermixing of strains among diverse animal hosts, likely through the environment, and thus a more integrated, “One Health” type of approach to addressing this issue is needed (Ewers et al., 2012). Because of the animal component, animal health interventions that target APEC are of the utmost importance. For example, improvements in APEC vaccine efficacy (Nordstrom et al., 2013) would aid human health in multiple ways. First, if there is some connection between APEC and human UTIs, a poultry vaccine would therefore clearly reduce human illness. Second, reducing the burden of illness in poultry due to APEC would also reduce the need for antibiotic treatment of sick flocks, and this reduced antibiotic usage would have a positive result for both human and animal health. Models have been developed that assess the relationship between animal health and human health (Singer et al., 2007), and these types of risk-benefit models need to be expanded into ecologically-based risk assessments (Ashbolt et al., 2013). To reduce the burden of illness associated with ExPEC, the scientific community needs to push forward with ecologically-based, scientifically-sound study designs that can address the plethora of ways in which *E. coli* can spread. Labeling UTIs caused by ExPEC as FUTI does nothing to further this mission and distracts from the holistic approach required to mitigate this serious global burden of illness.

### Conflict of Interest Statement

Dr. Randall S. Singer has previously consulted with Zoetis, Elanco Animal Health, Bayer Animal Health and the Animal Health Institute. The research for this manuscript was conducted in the absence of any commercial or financial relationships that could be construed as a potential conflict of interest.
